# Nondestructive and intuitive determination of circadian chlorophyll rhythms in soybean leaves using multispectral imaging

**DOI:** 10.1038/srep11108

**Published:** 2015-06-10

**Authors:** Wen-Juan Pan, Xia Wang, Yong-Ren Deng, Jia-Hang Li, Wei Chen, John Y. Chiang, Jian-Bo Yang, Lei Zheng

**Affiliations:** 1 School of Biotechnology and Food Engineering, Hefei University of Technology, Hefei 230009, China; 2 School of Medical Engineering, Hefei University of Technology, Hefei 230009, China; 3 Department of Computer Science and Engineering, National Sun Yat-sen University, Kaohsiung 80424, Taiwan; 4 Department of Healthcare Administration and Medical Informatics, Kaohsiung 80708, Taiwan; 5 Rice Research Institute, Anhui Academy of Agricultural Sciences, Hefei 230031, China

## Abstract

The circadian clock, synchronized by daily cyclic environmental cues, regulates diverse aspects of plant growth and development and increases plant fitness. Even though much is known regarding the molecular mechanism of circadian clock, it remains challenging to quantify the temporal variation of major photosynthesis products as well as their metabolic output in higher plants in a real-time, nondestructive and intuitive manner. In order to reveal the spatial-temporal scenarios of photosynthesis and yield formation regulated by circadian clock, multispectral imaging technique has been employed for nondestructive determination of circadian chlorophyll rhythms in soybean leaves. By utilizing partial least square regression analysis, the determination coefficients R^2^, 0.9483 for chlorophyll a and 0.8906 for chlorophyll b, were reached, respectively. The predicted chlorophyll contents extracted from multispectral data showed an approximately 24-h rhythm which could be entrained by external light conditions, consistent with the chlorophyll contents measured by chemical analyses. Visualization of chlorophyll map in each pixel offers an effective way to analyse spatial-temporal distribution of chlorophyll. Our results revealed the potentiality of multispectral imaging as a feasible nondestructive universal assay for examining clock function and robustness, as well as monitoring chlorophyll a and b and other biochemical components in plants.

Circadian clock, an endogenously driven regulatory mechanism that synchronized with the external diurnal light/dark cycles, generates periodic physiological, biochemical and developmental processes for plants to anticipate recurring daily changes and confer a fitness advantage^1^,^2^,^3^,^4^,^5^,^6^. The well-studied core oscillator of circadian clock in *Arabidopsis thaliana* is formed by the main transcription-translation feedback loop composed of the oscillator CIRCADIAN CLOCK ASSOCIATED1 (CCA1), TIMING OF CAB EXPRESSION1 (TOC1) and LATE ELONGATED HYPOCOTYL (LHY)^7^,^8^,^9^,^10^,^11^. Controlled by a circadian clock, plants maximize growth and survive in an intricate genetic network which controls periodic developmental units in response to environmental alterations and endogenous signals such as day length, temperature, hormonal status, essential nutrients and water^12^,^13^,^14^,^15^. When timed correctly, the transition, an important phase change from vegetative to reproductive development in a plant’s life, helps to ensure reproductive success and adaptive value. With dysfunctional clocks, bad timing decreases visible leaf area, total chlorophyll and net photosynthesis, and this in turn reduces biomass formation/accumulation and yield^16^,^17^,^18^,^19^. Stress assessment also proved that plants with an endogenous clock period matched to the environment cues enhance fitness and survive better than plants with circadian periods failing to keep pace with their environment by improving their ability to adapt to extrinsic influences^20^,^21^,^22^,^23^. To uncover the crucial role that endogenous circadian clock plays in plants fitness, it is important to develop a simple, nondestructive, real-time and intuitive approach for the measurement of circadian rhythms.

In order to tackle this problem, a bioluminescence approach based on luciferase (LUC) reporter fusions has been developed, requiring the insertion of promoter: LUC fusions into plants genome for revealing rhythmical patterns of gene expression, and laid a good foundation for a better understanding on the temporal regulation of circadian rhythms outputs and the correlation between clock function and performance in crops^24^,^25^,^26^,^27^. However, the important drawbacks for this method are: (i) invasion, costliness and time-consuming due to an insertion technique; and (ii) short monitoring time due to signal attenuation caused by gene degradation. These factors have limited application of this method to high-throughput analyses of circadian clock.

More recently, Gould *et al.*^18^ have utilized a delayed fluorescence (DF) approach to measure the rhythmicity of chlorophyll accumulated amount, indicating that chlorophyll accumulated amount can be used as a potential indicator of endogenous circadian. In this system, DF is a long-lived light emission by a plant based on the recombination fluorescence, which does not require genetic modification and thus has a wide range of suitability. However, the disadvantages for delayed fluorescence method are: (i) only components with active recombination fluorescence are detectible; (ii) a highly sensitive detector due to the weak signal; and (iii) capture luminescent image immediately after illumination due to signal rapidly decays within 50 second which might influence rhythmicity. Compared to the delayed fluorescence approach, multispectral imaging (MSI) approach is a simpler assay, which has several distinct advantages. MSI was developed by integrating the imaging and spectroscopy techniques together, which makes it possible to acquire both spatial and spectral information from a target object simultaneously. MSI with 19 different spectral bands has an excellent ability to determinate the spatial-spectral signature, especially for characterizing a variety of chemical composition and assessing physiological status of plants^28^,^29^. Continuous automated monitoring of dynamic spatial variation, showing how circadian clock allows plant to optimize its growth and development and in turn increase yield formation, is a unique advantage of imaging^30^,^31^,^32^. MSI used to study spatial variation over time can contribute to improving our understanding of the regulation of circadian rhythms and the mechanisms of coordinated stomata responses to changes in environmental conditions^33^. Furthermore, MSI with nondestructive advantages is of great value for applications such as remote analyzing the physiological status of a plant and determining the extent to which plant is stressed or at risk from environmental disaster.

Chlorophyll is the predominant molecule used in plants to capture light energy, which is subsequently used in the process of photosynthesis to convert carbon dioxide and water into organic compounds. NASA researchers have used orbiting carbon observatory–2 (OCO–2) as a new tool to successfully monitor the “glow” of the chlorophyll (“signatures” of photosynthesis) contained within plants, a phenomenon known as solar-induced chlorophyll fluorescence, opening up potential new applications for studying vegetation on land (http://www.nasa.gov/press/2014/april/nasas-carbon-counting-spacecraft -arrives-at-launch-site/#.U31YOXAZSoA). Recent studies have revealed that there are circadian rhythms in transcript abundance of genes associated with synthesis and accumulation of chlorophyll, and related proteins^34^. The output rhythmic control exerted on *Lhcb* genes is one of the most carefully studied examples. *Lhcb* genes—Lhcb1*1 and Lhcb1*3 (also called *CAB2* and *CAB1*) encode the most abundant protein in photosynthetic membranes comprising up to 50% of the thylakoid membrane protein that binds up to 50% of the chlorophyll in plants^34^. Light-harvesting chlorophyll a/b proteins and apoprotein synthesis are known to be tightly coordinated with chlorophyll biosynthesis and several *cis*-acting sequence elements important for light-induced transcription and circadian control of these genes have been identified^35^,^36^,^37^,^38^. Previous reports indicated that correct matching of the circadian period with the external one increases chlorophyll accumulation, which confirmed the dependence of chlorophyll accumulation on clock function^16^. Thence, the circadian rhythm of chlorophyll accumulated amount may be a potential indicator of endogenous circadian rhythm related to photosynthesis.

Although present knowledge of the circadian clock suggests conservation among plant species, studies of clock function and molecular architecture in plants other than *Arabidopsis thaliana* are limited^39^,^40^. For instance, between the circadian clock and responses to environment, there is a well-established regulatory link in model plants such as *Arabidopsis thaliana* while little is known of the circadian system in other crop species like soybean (one of the four major food crops). Soybean is an otherwise well-studied crop in terms of its genetics and related molecular behavior except for the behavior of circadian oscillators. In this article, we describe the applicability of MSI technique for quantitative measurement of chlorophyll accumulated amount in the intact soybean leaves. We report that this approach can also be applied to identification of circadian chlorophyll rhythm in wheat and moss Physcomitrella patens. Ultimately, our results demonstrate that MSI offers a simple, nondestructive, and real-time monitoring universal assay for examining clock function and robustness, as well as monitoring spatial-temporal distribution of chlorophyll or many other chemical compositions in plants.

## Results

### Circadian modulation of chlorophyll signaling

**Figure 1:**
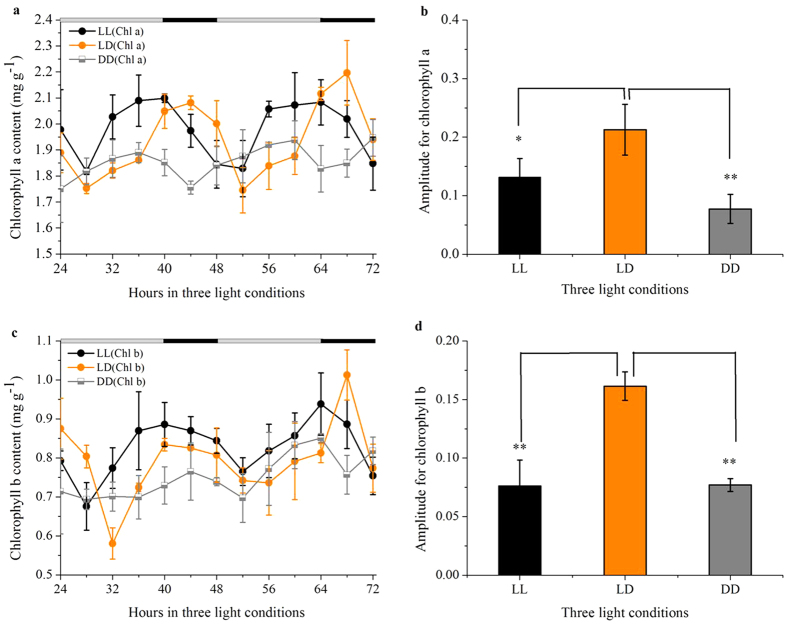
Measured chlorophyll a (a) and b (c) concentrations showed rhythmic activities under LD, LL, and DD conditions. Amplitude for chlorophyll a (**b**) and b (**d**) in three light conditions was calculated by FFT-NLLS analysis according to data from ZT24 to ZT72. Data are means ± SEM of n = 15 soybean leaves from three independent experiments.

How the circadian clock exercises its control over chlorophyll accumulation with 24 h light/dark cycle, however, is poorly understood. To test if chlorophyll accumulated amount can be used as a potential indicator of endogenous circadian rhythm related to photosynthesis, we further analyzed the variations of chlorophyll concentrations in soybean leaves over typical 24 h light/dark cycles. As shown in Fig. 1, there was significant change in chlorophyll concentrations throughout the 48 h period. The chlorophyll a and b concentrations exhibited much more visible circadian amplitude with sharp peak and very low baseline under LL than under DD (Fig. 1a,c). The diurnal oscillations under LD exhibited much more visible amplitude than circadian oscillations under LL and DD (Fig. 1b,d). For this study, chlorophyll a and b concentrations exhibited a rhythm under LD and LL conditions. However, disparate matching of endogenous rhythms to environmental rhythms under DD reduced leaf chlorophyll content. On examining the phase alteration, a 6.1-h and 1.9-h advanced accumulation phase were observed for chlorophyll a (Fig. 1a and Table 1) and chlorophyll b (Fig. 1c and Table 1) under LL with respect to LD conditions, respectively. Of note, important differences are observed for chlorophyll b contents under LL and LD during the first 40 h. As we all known, the increase in chlorophyll b content was accompanied by enhanced chlorophyllide a oxygenase (CAO) and Lhcb1*1 gene expression^41^. Previous reports strongly support the notion that regulation in the rate of gene transcription by irradiance is a primary determinant in the abundance of CAO and Lhcb1*1 mRNA^42^. It has been proposed that transcription of both CAO and Lhcb1*1 genes decreased to a very low level within 1.5 h after a low irradiance light(LIL)→high irradiance light (HIL) shift. Once soybean seedlings were transferred from dark to light conditions, decreased CAO and Lhcb1*1 gene expression lead to lower chlorophyll b than no irradiance changes.

Overall, our data indicate that diurnal cycle is required for maintaining the phase (Table 1), amplitude (Fig. 1b,d) and period length (Table 1) of chlorophyll content. Previous studies indicated that many of the key regulators in circadian control exhibit light-regulated expression characteristics^9^.

### Prediction for circadian rhythms of chlorophyll concentration in soybean leaves

**Figure 2:**
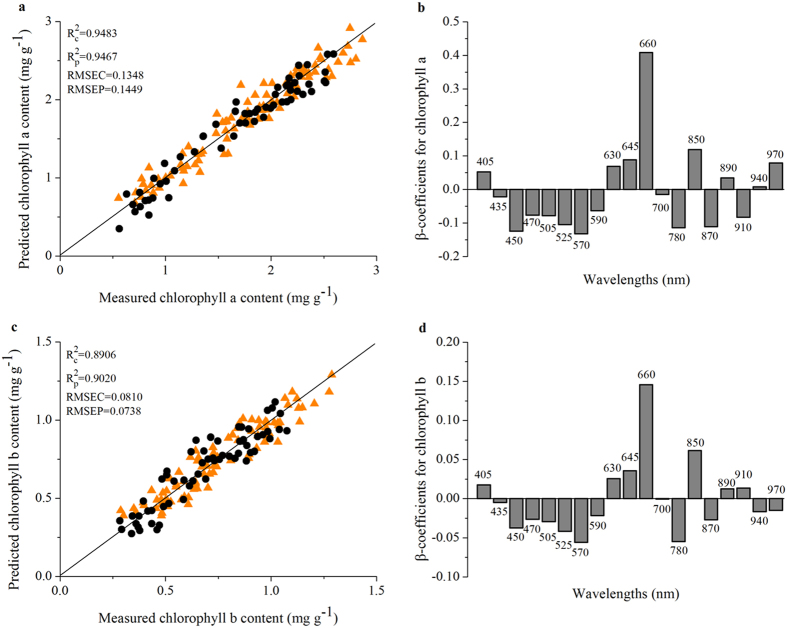
Measured and predicted values of chlorophyll a (a) and b (c) concentration for the PLSR models under the full range spectra. 90 samples (orange triangle) were used for training set and 60 samples (black dot) were used for test set. Regression coefficients of the PLSR model for chlorophyll a and b parameter are shown in (**b**) and (**d**), respectively. Three independent experiments were performed with similar results.

Since the sRGB images and reflectance value of soybean leaves at different ages are different from each other (Fig.S1a–c), we next examined the average reflectance value at 4 h intervals for a total of 48 h at different spectral bands variations and calculated the chlorophyll concentrations in soybean leaves under each light condition (Fig. S1d). For this study, we first extracted reflectance spectrum data (see supplemental information section about spectral data extraction details) and connect the average spectrum value of soybean leaves with chlorophyll concentrations in order to establish calculated model in which 150 samples were examined. 90 samples were used for training set and 60 samples were used for test set. The average spectrum values of soybean leaves and the corresponding chlorophyll a and b concentrations were set as input data for partial least squares regression (PLSR, see supplemental information section about PLSR calibration models details). The prediction results are shown in Fig. 2.

Our models predict chlorophyll a and b with the determination coefficients R^2^ of 0.9483 and 0.8906, respectively (Fig. 2a,c). These characteristics are directly related to the optical reflectance properties of the soybean leaves. Also, the PLSR predicted values for chlorophyll a and b maintained substantially lower deviations across the entire range of their reference values with RMSEP (the root mean square error of prediction) of 0.1449 and 0.0738, respectively. Previous reports indicated that the accuracy of regression model is evaluated as being excellent when the R^2^ is 0.8 or higher^43^. These models have good prediction ability as revealed by higher value of R^2^ as well as smaller values of RMSEV (the root mean square error of validation) and RMSEP (RMSEP < RMSEV). The model performance in terms of R^2^, RMSEV and RMSEP, implies that PLSR is adequate for the prediction of the chlorophyll a and b concentrations.

Actually, our data also can be established based on the full range spectra and β-coefficients resulting from PLSR models for chlorophyll a and b, respectively (Fig. 2b,d). Wavelengths corresponding to the highest absolute values of β-coefficients, 450, 525, 570, 660, 780, 850 and 870 nm was identified as important wavelengths. The other eleven wavelengths were excluded as the corresponding noise (such as a low signal-to-noise ratio).

**Figure 3:**
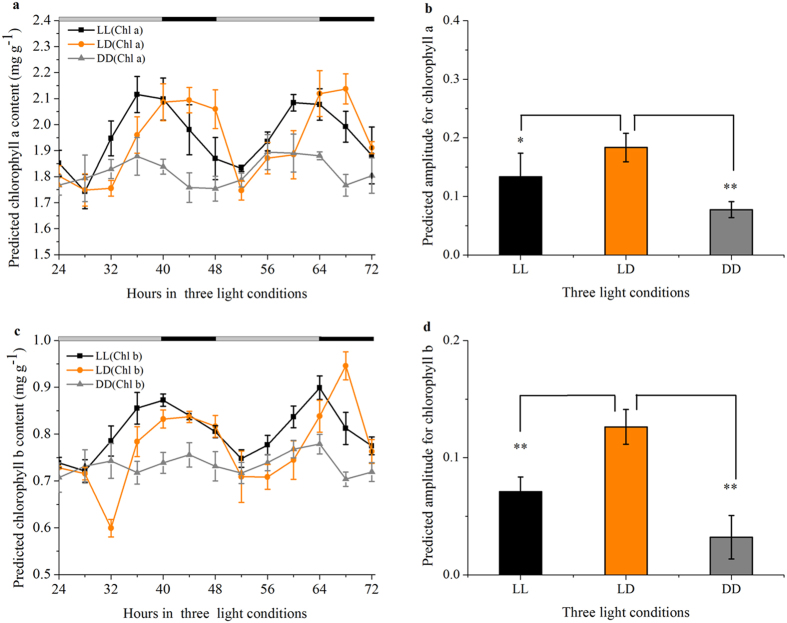
Circadian rhythms of predicted chlorophyll a (a) and b (b) concentrations based on PLSR functions when they were exposed to LD, LL, and DD conditions. Amplitude for chlorophyll a (**b**) and b (**d**) in three light conditions was calculated by FFT-NLLS analysis according to data from ZT24 to ZT72. Data are means ± SEM of n = 15 soybean leaves from three independent experiments.

Combined the PLSR functions derived for chlorophyll a and b on the full range spectra with the variations of spectral value during a 24-h period under LL, LD and DD conditions, we next calculated the variations of chlorophyll concentrations in soybean leaf blades over typical 24 h light/dark cycles, as shown in Fig. 3. The predicted level of chlorophyll a (Fig. 3a) and b (Fig. 3c) are under robust and accurate circadian control. However, chlorophyll a and b did not exhibit a rhythmic accumulation pattern with incorrect matching of endogenous rhythms to environmental rhythms under DD, causing the amplitude to be relatively flat (Fig. 3b,d). A 4.5-h and 2-h advanced accumulation phase were seen for chlorophyll a and b under LL than under LD conditions, respectively, and this was consistent with the actual measured value (Fig. 1a,c). The mean concentration of chlorophyll a is indistinguishable between the actual measured value and the predicted value while there is a little difference for chlorophyll b.

But using this method then, it is shown that predicted chlorophyll a (Fig. 3a) and b (Fig. 3c) of periodicity can be observed from the data, which might be good enough to characterize a circadian rhythm, in order to investigate clock function. What is clear from our results is that the prediction for circadian rhythm of chlorophyll concentration in soybean leaves is evaluated as being excellent, but PLSR provides significantly larger numbers of variables and is not a time-efficient method to enable fast and simple screening of large numbers of plant cultivars with dysfunctional clocks. Therefore, designing a simple calibration algorithm with a minimal redundancy is an issue in real applications.

### Real-time monitoring of circadian rhythms under optimal wavelength

**Figure 4:**
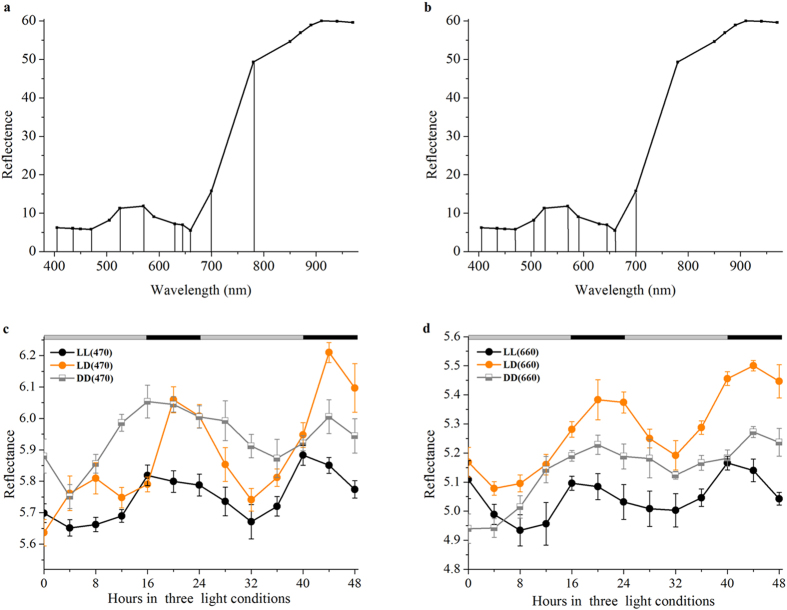
Circadian rhythms of the reflectance value under the optimal wavelengths selected for chlorophyll a and b by SPA. Plot of ten wavelength variables for chlorophyll a (**a**) and b (**b**) selected by SPA. Columns represent selected optimal wavelength variables. Black curve shows original spectrum. Circadian rhythms of reflectance value at 470 nm (**c**) and 660 nm (**d**) under LL conditions. Data are means ± SEM from three independent experiments.

The proper mean, which is developed to enable fast and simple screening of large numbers of plant cultivars with dysfunctional clocks, is crucial for the non-destructive measurement of rhythmic patterns in plant growth. More recently, successive projections algorithm (SPA) process for the informative variable selection was found to be efficient, because the optimal wavelengths selected by SPA have fewer number of variables and have less co-linearity than full range spectra^43^. In the following, we sought to select the optimal wavelengths for chlorophyll a and b in order to confirm the circadian rhythm of chlorophyll concentration in soybean leaves and the result was illustrated in Fig. 4. Ten variables (405, 435, 470, 525, 570, 630, 645, 660, 700 and 780 nm) were selected as the optimal wavelengths which could later be used to predict the chlorophyll a content of soybean leaves (Fig. 4a). By following the same fashion, ten variables (405, 435, 470, 505, 525, 570, 590, 645, 660 and 700 nm) were selected as the optimal wavelengths to predict the chlorophyll b content in soybean leaves (Fig. 4b). Large variations in the visible spectrum range of 405–780 nm were observed while reflectance was relatively flat over the spectral region of 780–970 nm (Fig. 4a,b). Two distinctive downward peaks were observed around 470 nm and 660 nm mainly due to chlorophyll absorption. This result was consistent with previous report that peaks at 428–760 nm were due to the color changing^44^. As pointed out by a substantial body of publications, the wavelength reflectance near 550, 670, 700 and 708 nm has been found to be highly sensitive to chlorophyll a concentration^45^,^46^. A number of other studies have found that the reflectance around near-infrared range of the spectrum (above 750 nm) to that 690–730 nm were well correlated with the total chlorophyll content of leaves^47^,^48^.

The next question is whether the chlorophyll absorption peaks in 470 nm and 660 nm are under the control of central oscillators. To answer this, we tested the circadian rhythms in the chlorophyll absorption peaks. The reflectance exhibited a robust rhythm under LD and LL conditions (Fig. 4c,d). Under LD conditions the soybean leaves exhibited high-amplitude rhythms with sharp peaks at the transition of subjective night to subjective day, and a 4-h advanced reflectance phase was both observed for 470 nm and 660 nm under LL than under LD conditions (Fig. 4c,d).These observations are consistent with clock regulation of the accumulation of chlorophyll a and b. The similarity in the period of oscillation for the reflectance of chlorophyll absorption peaks in 470 nm and 660 nm with circadian rhythms of chlorophyll a (Fig. 1a) and chlorophyll b (Fig. 1c) concentrations supports the hypothesis that reflectance rhythms are driven by the central oscillator. Therefore, these results demonstrate that soybean leaves in chlorophyll absorption peaks exhibited an endogenous oscillation of reflectance that correlated well with the circadian fluctuation of the intracellular level of chlorophyll accumulation. So this assay can be advantageously used for continuous circadian plant monitoring, both to visualize whole plant responses and to follow the responses of individual leaves. Simultaneously, we can measure reflectance rhythms in a larger number of single soybean seedlings or groups of soybean seedling, making the assay high-throughput screening of large numbers of plant cultivars for their response to circadian fluctuation. And it would be particularly suitable for studies such as screening of plant lines engineered for increased photosynthesis.

### Plant rhythms monitoring and time-lapse imaging

**Figure 5:**
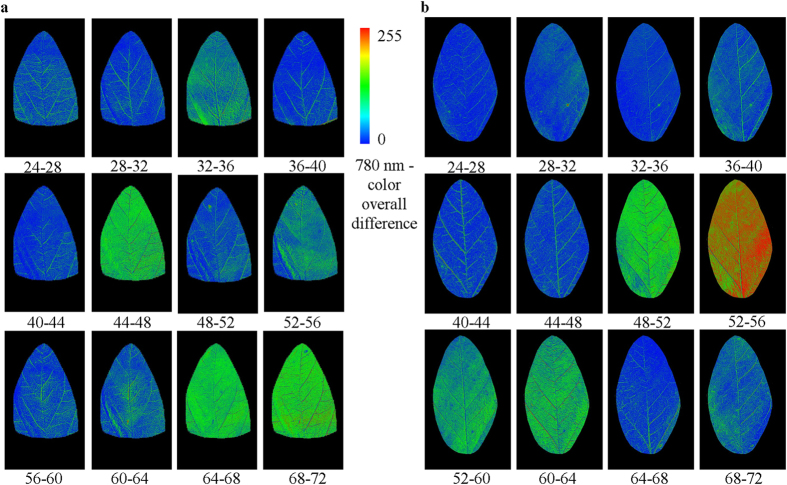
Reflectance differential images (color) exhibit the rhythm of heterogeneity at 780 nm during the recordings of Fig. 4 for soybean leaves in LL condition (a) at different time in addition to DD (b).

Since the reflectance rhythms could be coupled with the chlorophyll rhythms controlled by the central oscillators, the next question is whether reflectance imaging also exhibits the circadian rhythms in chlorophyll characteristic spectral bands. To test for circadian rhythms in reflectance imaging, the differential analytical method was utilized to analyze these imaging at different time points, and the results are shown in Fig. 5 and supporting information Fig. S2.

The reflectance images (Fig. 5 and supporting information Figs S2, S3) demonstrate that there was heterogeneity over the leaves during the oscillations of light/dark cycle under the three light conditions. Except for the DD condition (Fig. 5b and supporting information S3b), time-lapse imaging shows that the spatial structure of heterogeneity under LL condition (Fig. 5a and supporting information S3a) and LD condition (not shown) showed circadian oscillation rhythm between homogeneous and heterogeneous states. This is consistent with the chlorophyll circadian rhythms. The differential images of soybean leaves, reaching the highest heterogeneity degree at 20–24 h and 44–48 h (Fig. 5a), may imply that heterogeneity is rhythmic.

Multispectral reflectance imaging, the only imaging system that can be used to study spatial variation over time, is applied from the microscopic scale to remote sensing, and contributes to improving our understanding of the regulation of circadian rhythms and the responses mechanisms to changes in environmental conditions. Continuous surveillance of visual images can help us to observe plant growth rhythms fluctuating with the external light/dark cycles. Dynamic fluctuations of plant growth reflect adjustments of endogenous processes to variations of environmental conditions whose elucidation is vital to the understanding of biomass accumulation and yield.

**Figure 6:**
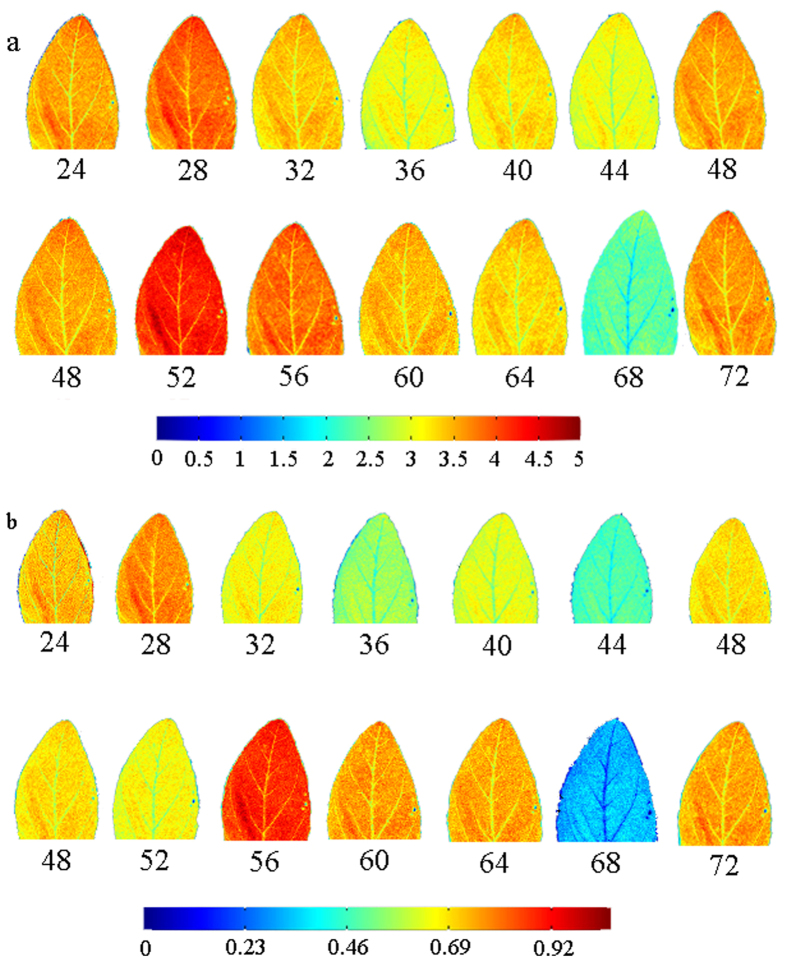
Visualization maps for chlorophyll a (a) and b (b) content distribution of soybean under LL condition. The parallel color bar represents the chlorophyll content of the images.

As shown in Fig. 6, the maps of chlorophyll a (Fig. 6a) and b (Fig. 6b) distribution in soybean leaves under LL conditions were produced by applying PLSR model at 19 wavelengths (range from 405 nm to 970 nm) to the multispectral images. The levels of chlorophyll concentration, from high (red) to low (blue), were shown in different colors (Fig. 6). The spatial and concentration distribution of chlorophyll in these leaves (Fig. 6) could be visualized via pixel analysis of multispectral images and inserting processing. Obviously, these maps help us to understand the change of chlorophyll concentration in soybean leaves during different time point. Imaging shows that the spatial and concentration distribution of chlorophyll a (Fig. 6a) and b (Fig. 6b) under LL condition and LD condition (not shown) showed circadian oscillation rhythm. Thence, visualization of chlorophyll or other nutrients will be an important direction in the future research for the evaluation of the circadian in plants.

### Circadian rhythms can be measured by MSI in stress conditions and a range of plant species

**Figure 7:**
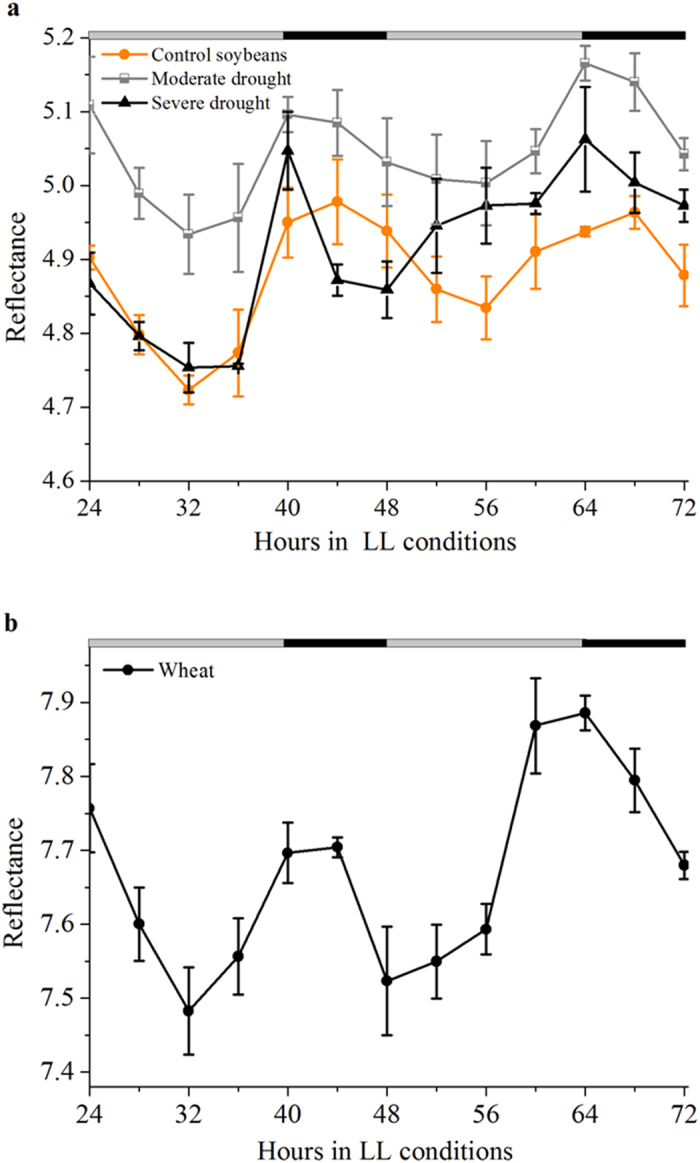
Circadian rhythms can be measured by MSI under stress conditions and with a range of plant species. (**a**) Soybean plants under drought stress condition. (**b**) Wheat plants.

With a diverse array of strategies, plants maximize growth and survive in a world of frequent environmental alterations imposed by the natural day/night cycle and variable weather conditions to achieve maximal growth efficiency. Stress conditions (examples: long-term soil water deficit, temperature or iron deficiency) are the primary limitation on plant growth, photosynthesis and yield of crops in agricultural systems^49^. In order to highlight the uses of the MSI approach, we investigated whether the reflectance imaging is suitable to measure plant stress due to drought conditions (Fig. 7a). Soybeans exposed to the moderate and severe drought stress conditions showed significant changes in the diurnal reflectance value with respect to normal well-watered conditions. The moderate stress condition did not affect chlorophyll circadian rhythms (cycle length and amplitude), whereas higher amplitude of reflectance value was observed 4 hours earlier under severe stress conditions (Fig. 7a).The lower amplitude of reflectance value also displayed advanced-phase expression under severe hydration stress, correlating well with previous studies that drought stress conditions modify the regulation of soybean LHY/CCA1-like gene expression. To examine whether MSI approach is unique to soybean or whether it could be observed in wheat and the moss Physcomitrella patens. Our data suggest that MSI approach were also robust to measure the circadian rhythm of wheat, not including the moss Physcomitrella patens (Fig. 7b and supporting information Fig. S4). The MSI approach may lead to potential new applications for studying the circadian rhythm of plants, but more extensive research on the use of this assay in relation to clock related processes is needed, to improve reliability of the method. For the applicability to bryophytes extensive research is still necessary, as the rhythm observed in the moss Physcomitrella patens was rather faint (Fig. S4).

## Discussion

Environmental light signals, the most effective signals in synchronizing the environment cue and the internal circadian clock in plants, act as severe instructors that play a crucial role in resetting the circadian clock and promoting plants to evolve highly complex sensory mechanisms to monitor surroundings and adapt growth and development for optimal rhythm to the prevailing environmental conditions. Photosynthesis has a marked effect on the entrainment and maintenance of circadian rhythms in *Arabidopsis thaliana*^50^. Current models for the chlorophyll content oscillations indicate that leaves of etiolated seedlings exposed concomitantly to continuous light, and show a propensity for accumulating chlorophyll amounts which oscillates according to the time of day. However, due to the damage of plant growth, time-consuming and complexity, only low-throughput approach exists for measuring circadian rhythms. These observations prompted us to search for a promising tool used for non-destructive plant monitoring and screening to measure period, robustness and accuracy of the circadian clock.

According to previous study, leaf reflectance imaging in different spectral bands has proven to represent an effective tool for non-destructive plant monitoring (including circadian rhythms monitoring) and a ‘road’ leading to a broad range of identification and screening tasks^29^. It has become obvious that the rhythm of soybean leaves in the chlorophyll absorption peaks (Fig. 4c,d) correlates well with the circadian expression pattern of the chlorophyll concentrations (Fig. 1). This is the first time to ‘live’ monitor the changes of chlorophyll a and b accumulation using a non-destructive method. Thus, the result unveils the potential of MSI system as a non-destructive and intuitive method of assaying central clock function in higher photosynthetic organisms.

It is particularly noteworthy that there is a good correlation between the phase of multispectral reflectance rhythm in soybean leaves and chloroplast psbD light-responsive promoter gene transcript rhythm^51^. The close correlation between the actual measured (Fig. 1) and the predicted chlorophyll rhythm (Fig. 3) in this study suggests that the multispectral reflectance rhythm is a reliable readout of the same oscillator that drives chloroplast gene expression. Harmer *et al.*^52^ support the notion that virtually all clock-controlled genes associated with chlorophyll synthesis and the light-harvesting apparatus exhibit peak circadian transcript abundance 4 hours after subjective dawn, and this time coincided with the increasing trend of chlorophyll synthesis in ZT4 and ZT28 (Fig. 1a,c). The idea that a shorter period and an advanced phase of Lhcb1*1 circadian expression due to *HY5* - a positive regulator of photomorphogenesis (the control of plant development by light) in *hy5* mutant seedlings^37^. Our 4-h advanced accumulation of chlorophyll circadian accumulation phase results is the same as the advanced expression phase of Lhcb1*1 circadian, showing that LL conditions may lead to changes in *hy5* gene expression level. Taken together, recent studies have demonstrated a correlation between the central circadian clock and transcript genes associated with chlorophyll synthesis, suggesting a close connection between both pathways. Chlorophyll accumulation rhythms (Fig. 1a) are similar to the reflectance imaging rhythms in specific wavelength such as 660 nm (Fig. 4d), suggesting the reflectance method should provide a simple tool for plant rhythm monitoring. In addition to chlorophyll a and chlorophyll b, the multispectral reflectance imaging approach, unlike DF, also can be used for other nutrients monitoring, such as water, carbohydrate^18^.

This study provides a strong foundation for further studies investigating the matching of endogenous rhythms to environmental rhythms and the effect on plant growth and fitness, and opens up the possibility to use this to optimize photosynthesis and overall productivity^53^,^54^. Correlating the real-time monitoring with the plants yield and performance will be essential to ensure an optimal phase relation between physiology and the day/night cycle. It will be worthwhile in anticipation of future changes so as to maximize growth and survive in a world of frequent environmental alterations in plants. Here, we have shown that leaf reflectance in different spectral bands can be used as a powerful tool to measure rhythms and investigate the correlation between clock function and performance in crops. As a circadian time-supervisor, the MSI method has many potential advantages over current methods for assaying clock function. For plants that are difficult to cross or transform, the insertion of a transgenic used for assay clock function is time-consuming (about 4–6 months or even longer), and this drawback greatly limits the industrial application by crossing or transformation. The MSI approach is a non-destructive and intuitive approach to real-time screen the plant rhythms, while allowing measurement of rhythms in natural soil–plant systems when operated under strict environmental control. In addition to strict drought environment, MSI approach was suitable in the wheat, including not the moss Physcomitrella patens due to faint rhythmic (Fig. S4). Moreover, previous studies have reported that the regulatory link between the clock and *Lhcb* genes in the moss Physcomitrella patens shows characteristics that appear to differ from those in higher plants^55^. The study will open up potential new applications for studying various plant secondary metabolites, leading to a better understanding of the dynamic kinetic of plant growth, biomass process and yield formation. Also, the nondestructive and direct image observation advantages of MSI analyses makes it an ideal candidate for remotely screening and revealing the dynamic patterns of plant-environment interactions that underlie the adaptability of plant to a wide variety of environment.

## Methods

### Plants material and growth conditions

Freshly soybean (Wandou 29) and wheat seeds were both obtained from Hefei Fengle Seed Co., Ltd (Hefei, China). The mature soybean and wheat seeds were tested for viability prior to the germination tests, and the rate of germination is more than 98%. The light intensity was measured by an optical power test instrument (SGN-II), manufactured by Tianjin Gangdong Sci. & Tech. Development Co., Ltd (Tianjin, China)^56^. Mature seeds were kept at 4 °C in the dark for 3 days to synchronize the germination, and then grown directly in soil under 16 h light: 8 h dark cycles conditions at a light intensity of 200 μW for 20 days (in order to fully entrain the circadian clock) in a plant growth room at 25 ± 1 °C before the leaves were harvested. After the fifth true leaf was developed (about 20 days after germination), twenty-day-old soybean and wheat seedlings (Fig. S1e,f) grown under 16 h of light and 8 h of dark were transferred to free-running conditions of LL (continuous light, time 0), DD (continuous dark, time 0) and LD (16 h light/8 h dark cycle, time 0) for 24 hours before harvesting commenced. Time points were taken every 4 h across the 48-h experiment for chlorophyll extraction and analyses after 24 h of free-running time. The variations of chlorophyll concentrations and the reflectance value at different spectral bands in soybean leaf blades over typical 24 h light/dark cycles were examined. The soil moisture was calculated by the gravimetric humidity (70% for control treatment: 30% for moderate stress treatment: 15% GH for the severe stress treatment), which corresponds to the percentage of water in the soil in relation to the dry weight of the soil. The moss Physcomitrella patens (Fig. S1g) were grown on BCDA medium. The choice of 16 h light: 8 h dark cycles was based on the work of Marcolino-Gomes *et al.*^40^. The selection of a 48 h time period (from 24–72 h after the initiation of LL, LD and DD) was based on the work of Hudson^57^ for circadian analysis of soybean.

### Nutrition solution

The nutrition solution contains 5 mM KNO_3_, 2.5 mM KH_2_PO_4_, 2 mM MgSO_4_, 2 mM Ca(NO_3_)_2_, 0.05 mM Fe-EDTA, 0.07 mM H_3_BO_3_, 14 μM MnCl_2_, 0.5 μM CuSO_4_, 1 μM ZnSO_4_, 0.2 μM Na_2_MoO_4_, 10 μM NaCl and 0.01 μM CoCl_2_. Plants were grown individually in pots of sand watered with the nutrient solution every 7 days to stabilize pH value at 7.0.

### Selection of soybean leaves

The first to fifth true leaf equal to or greater than 4 cm long was harvested at 4 h intervals for a total of 48 h to examine the chlorophyll concentrations and the reflectance value at different spectral bands variations in soybean leaf blades over light/dark cues. Ten leaves were harvested for scoring for each biological replicate under every light condition (Fig. S1d).

### Chlorophyll extraction and analyses

Chlorophyll extraction and analyses were based on the work of Arnon^58^ on the absorption of light by aqueous acetone (80%) extracts of chlorophyll. Simultaneous equations with the specific absorption coefficients for chlorophyll a and b were used to calculate the content of chlorophyll a and b:


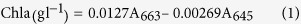



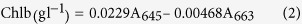


Acetone extracts of the samples were brought to 10 ml volumes, and chlorophyll a and b were measured spectrophotometrically at 663 and 645 nm on a UV-VIS spectrophometer (model 2802), produced by UNICO (Shanghai, China) instruments Co., Ltd.

### Multispectral imaging system

These leaves images were obtained by a MSI system with the VideometerLab equipment (Videometer A/S, Hørsholm, Denmark) at 19 different wavelengths (405, 435, 450, 470, 505, 525, 570, 590, 630, 645, 660, 700, 780, 850, 870, 890, 910, 940 and 970 nm) from the visual region to the lower wavelengths of the NIR region in the reflectance mode (Fig. S5). The MSI system mainly consisted of a high-resolution 1280 × 960, 45 μm monochrome grayscale CCD camera mounted in the top of the sphere, a specially assembled light unit consists of 19 high power LED sources with a range from 405 nm to 970 nm, along with one optional external light source, an integrating sphere with a matte white coating to ensure that the light is scattered evenly with a uniform, diffuse light at illumination. A computer equipped with a data acquisition and the VideometerLab software version 2.12.23 controls the exposure time, wavelength range, motor speed, image acquisition and image correction. The 19 light emitting diodes (LEDs) at the specific wavelength evenly distribute in the entire sphere rim and the soybean leave samples are placed inside the acquisition system to record surface reflections.

### Spectral data extraction and models analyses

The details of spectral data extraction, partial least square regression (PLSR) calibration and the successive projection algorithm (SPA) models are all available free of charge in supporting information. 90 samples were used for training set and 60 samples were used for test set.

## Additional Information

**How to cite this article**: Pan, W.-J. *et al.* Nondestructive and intuitive determination of circadian chlorophyll rhythms in soybean leaves using multispectral imaging. *Sci. Rep.* **5**, 11108; doi: 10.1038/srep11108 (2015).

## Supplementary Material

Supplementary Information

## Figures and Tables

**Table 1:** Time response for chlorophyll a and b reaching the peak or trough and period length were calculated by FFT-NLLS analysis according to data from ZT24 to ZT72. Phase difference between chemical analysis and the data predicted from the multispectral data in three light conditions was calculated. Three independent experiments were performed with similar results.

**Light conditions**	**The first peak**	**Trough**	**The second peak**	**Period length**
LL (Chl a/ Predicted Chl a)	37.2/37.8	49/50.4	61.6/61.8	24.4 ± 1.25/24.0 ± 0.57
LL (Chl b/ Predicted Chl b)	40.1/40	53.5/53	64.7/63.8	24.6 ± 1.3/23.8 ± 1.43
LD (Chl a/ Predicted Chl a)	43.3/42.3	53.4/54.3	67.4/66.6	24.1 ± 2.36/24.3 ± 1.61
LD (Chl b/ Predicted Chl b)	42/42	52/53.1	69.6/69.3	27.6 ± 2.17/27.3 ± 0.97
DD(Chl a/ Predicted Chl a)	34.7/34.8	44.9/45.2	57/57	22.3 ± 1.58/22.2 ± 2.03
DD (Chl b/ Predicted Chl b)	44.3/43.1	51.9/51.2	62/62.1	17.7 ± 0.56/19 ± 2.05
