# Supplementary material for: Nondestructive and intuitive determination of circadian chlorophyll rhythms in soybean leaves using multispectral imaging
Source: Sci Rep. 2015 Jun 10;5:11108. doi: 10.1038/srep11108 (PMC4461922; doi:10.1038/srep11108)
Supplement: Supplementary Information [file srep11108-s1.pdf]

Supporting information for

Nondestructive and intuitive determination of circadian chlorophyll rhythms in soybean leaves using multispectral imaging

Wen-Juan Pan [1], Xia Wang[2], Yong-Ren Deng[3], Jia-Hang Li[3], Wei Chen[1], John Y. Chiang[3,4], Jian-Bo Yang[5] & Lei Zheng[1, 2]

[1]School of Biotechnology and Food Engineering, Hefei University of Technology, Hefei 230009, China, [2]School of Medical Engineering, Hefei University of Technology, Hefei 230009, China, [3]Department of Computer Science and Engineering, National Sun Yat-sen University, Kaohsiung 80424, Taiwan, [4]Department of Healthcare Administration and Medical Informatics, Kaohsiung 80708, Taiwan, [5]Rice Research Institute, Anhui Academy of Agricultural Sciences, Hefei 230031, China.

Correspondence: Lei Zheng, Tel: 86-551-62919398. E-mail: lzheng@hfut.edu.cn, lei.zheng@aliyun.com

This supplemental information section includes the following:

Ten pages: spectral data extraction details, partial least squares regression (PLSR) calibration models details, optimal wavelength selection details, figures S1-S5, and reference

Spectral data extraction

The reflectance spectrum data, calculated by averaging the spectral value of all pixels in the ROI to produce only one mean value (spectrum), were extracted from solely the soybean leave regions by ignoring both the background and shadow. The final mask named 'Automatic Mask,' which is resulting from segmentation step, was used as the main ROI to extract spectral data from the calibrated multispectral image. The reflectance spectrum data, calculated by averaging the spectral value of all pixels in the ROI to produce only one mean value (spectrum), were extracted from solely the soybean leave regions by ignoring both the background and shadow. The same procedure was repeated to obtain the 19 mean spectrum values of other soybean leave samples from all multispectral images saved in a spectral excel.

Partial least squares regression (PLSR) calibration models

PLSR, a dimensionality reduction method, is emerging as the most robust and reliable chemometric method for constructing models and aims at determining predictor combinations with maximum covariance with the response variable when the measured variables are many and highly collinear[1-3]. The PLSR is an optimized version based on the linear algorithm, so the good calibration performance of PLSR for one response variable can be improved by eliminating of uninformative variables. The variable elimination is based on the PLSR coefficients β, the vector containing regression coefficients $\mathbf{b}$ ($K \times 1$), which are calculated by,

$$\beta = \mathbf{b} = \mathbf{W}\,(\mathbf{P}^{\mathrm{T}}\mathbf{W})^{-1}\mathbf{q}, \qquad \text{equation (1)}$$

Where $\mathbf{W}$ ($K \times A$) is the $\mathbf{X}$ weight matrix, $\mathbf{P}$ ($K \times A$) is an x-loading matrix and $\mathbf{q}$ ($1 \times A$) is the y-loading vector.

The PLSR model derived for chlorophyll a and b content on the full range spectra is shown as follow:

$$Y_{Chl} = \beta_0 + \sum_{i=1}^{i=n} X_i \times \beta_i \qquad \text{equation (2)}$$

Whereβ_0 and β_i are regression coefficients, Y_{Chl} is the measured chlorophyll a or b content of soybean leaves samples, X_i is the variable at the *i*th wavelength, n represents the number of wavelength.

In this study, the training set and the test set contain 90 spectra and 60 spectra, respectively. After that, PLSR was executed using MATLAB 7.11 (The Mathworks Inc., Natick, MA, USA). The performance of the final PLSR models is evaluated in terms root mean squared error of calibration (RMSEC) and the root mean squared error of prediction (RMSEP), as follows,

$$\mathrm{RMSEP} = \sqrt{\frac{\sum_{i=1}^{n}(y_i - \hat{y}_i)^2}{n}} \quad , \qquad \text{equation (3)}$$

$$R_p = \sqrt{1 - \frac{\sum_{i=1}^{n}(y_i - \hat{y}_i)^2}{\sum_{i=1}^{n}(y_i - \bar{y}_i)^2}} \quad , \qquad \text{equation (4)}$$

Where *n* is the number of samples in the test set. y_i , $\hat{y}_i$ are the reference measurement result and the estimated result of the model for the test set.

Optimal wavelength selection

The successive projections algorithm (SPA) is a variable selection technique designed to

solve collinearity effects by minimizing redundant variables in the training data set. The SPA process is disposed in a matrix **X** ($N\times K$) = ($x_1, \ldots, x_i$). Let **M** ($(N\text{-}1)\times K$)) be the maximum number of selected variables. The algorithm for construction of each chain starts from one of the variables x_k as follows,

$$Z^1 = x_k,\ for\ all\ \ k = 1, \ldots, K\ (the\ \ initial\ \ projected\ \ vectors\ )$$
$$X_j^{\,1} = x_j,\ for\ all\ j = 1, \ldots, K(the\ \ initial\ \ projected\ \ vectors\ )$$
$$SEL(1,\ k) = k$$
$$for\ all\ \ i = 1, \ldots, N$$
$$I = I\ (N\times N)$$
$$P^i = I - \frac{Z^i (Z^i)^T}{(Z^i)^T Z^i}(the\ \ projected\ \ matrix\ )$$
$$X_j^{\,i+1} = P^i X_j^{\,i}(the\ \ projected\ \ vectors\ )$$
$$j* = \arg\max_{j=1,\ldots,k} \left\| X_j^{\,i+1} \right\|(the\ \ lagerest\ \ projected\ \ vectors\ )$$
$$SEL(i+1, k) = j$$
$$Z^{i+1} = X_{j*}^{\,i+1}(the\ \ next\ \ iteration\ \ of\ \ the\ \ projection\ \ operation\ )$$

end

end

end

The algorithm was established based on previous studies[4-5], and the process of SPA, all figures analyses and statistics were carried out in Matlab 7.11 (The Mathworks Inc., Natick, MA, USA) and Origin 8.5.This is the first time to use SPA for the optimal spectral wavelength selection representing chlorophyll circadian clock.

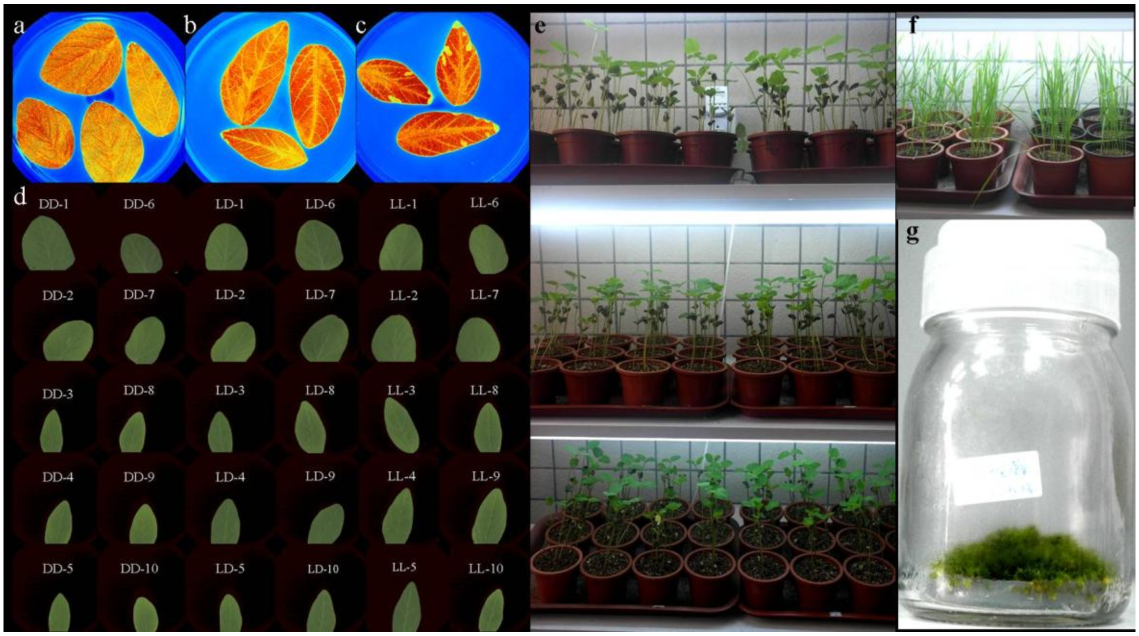

Supplemental figure S1 Circadian rhythms can be measured by MSI in stress conditions and a range of plant species. sRGB images of soybean leaves at 20 d (a), 30 d (b), 40 d (c) and ten leaves (d) were harvested for scoring for each biological replicate under LD, LL, and DD conditions. Soybean plants under three drought stress conditions (e). Wheat plants (f). The moss Physcomitrella patens (g).

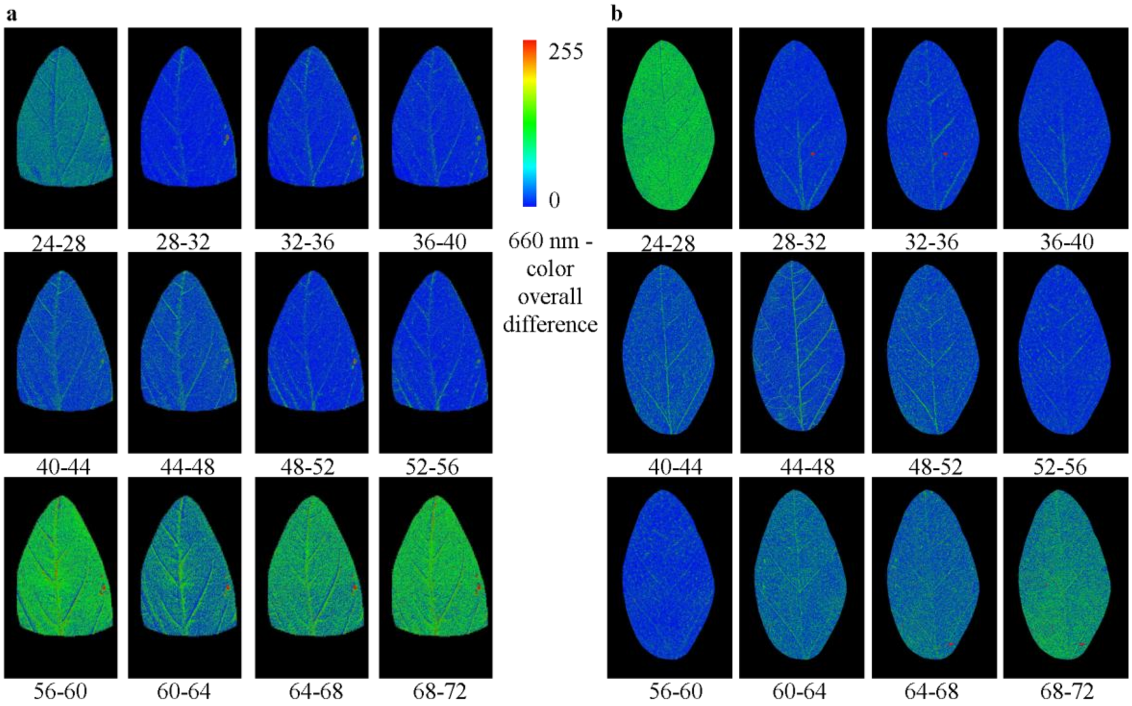

Supplemental figure S2. Reflectance differential images (color) exhibit the rhythm of heterogeneity at 660 nm during the recordings of Fig. 4 for soybean leaves in the LL conditions (a) at different time point in addition to DD conditions (b). Soybean seedlings were exposed to LL (continuous light) conditions and DD (continuous dark) conditions at 28 ℃. Twenty-day-old soybean seedlings grown under 16 h of light and 8 h of dark were transferred to LL (time 0) and DD(time 0). Soybean leaves images were captured by the imaging system at 4 h intervals for a total of 48 h.

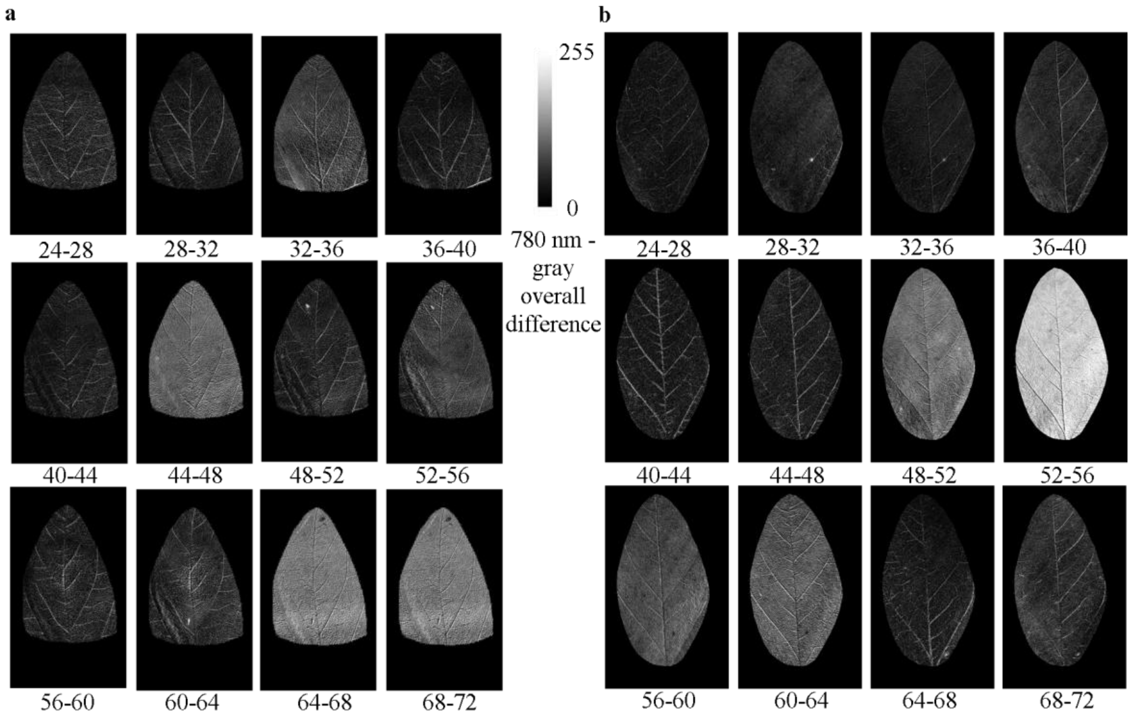

Supplemental figure S3. Reflectance differential images (gray) exhibit the rhythm of heterogeneity at 780 nm during the recordings of Fig. 4 for soybean leaves in the LL condition (a) at different time point in addition to DD (b).

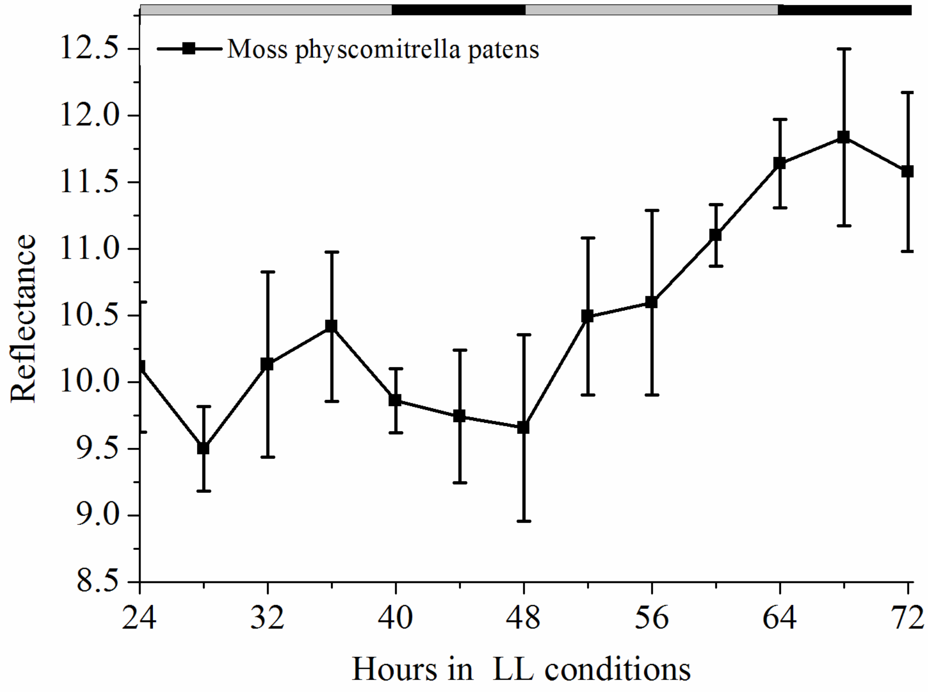

Supplemental figure S4. Circadian rhythms can be measured by MSI in the moss Physcomitrella patens.

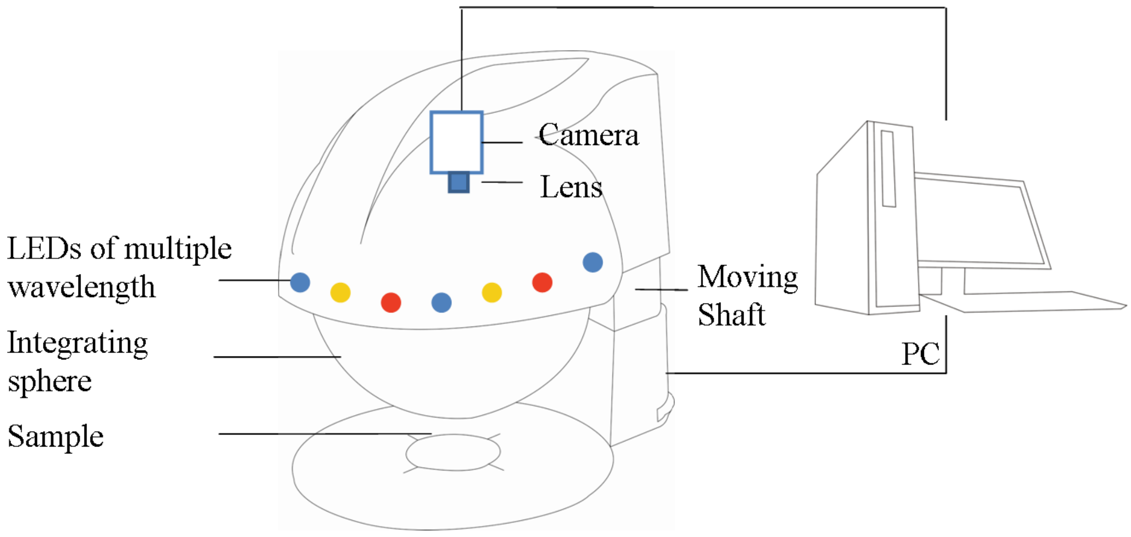

Supplemental figure S5. Principal setup of the multispectral imaging system. An integrating sphere with a matte white coating ensures optimal lighting conditions. The light emitting diodes located in the rim of the sphere ensures narrowband illumination. The image acquisition is performed by a monochrome grayscale CCD camera mounted in the top of the sphere.

1. Jungandreas, A., Wagner, H. & Wilhelm, C. Simultaneous measurement of the silicon content and physiological parameters by FTIR spectroscopy in diatoms with siliceous cell walls. *Plant Cell Physiol.* **53,** 2153-2162 (2012).

2. Couture, J. J., Serbin, S. P. & Townsend, P.A. Spectroscopic sensitivity of real-time, rapidly induced phytochemical change in response to damage. *New Phytol.* **198,** 311-319 (2013).

3. Sulpice, R. *et al*. Impact of the carbon and nitrogen supply on relationships and connectivity between metabolism and biomass in a broad panel of *Arabidopsis* accessions. *Plant Physiol.* **162,** 347-363 (2013).

4. Galvao, R. K. H. *et al*. A variable elimination method to improve the parsimony of MLR models using the successive projections algorithm. *Chemometr. Intell. Lab.* **92,** 83–91 (2008).

5. Wu, D. *et al*. Rapid prediction of moisture content of dehydrated prawns using online hyperspectral imaging system. *Anal. Chim. Acta* **726,** 57–66 (2012).
